# Spillover zoonotic ‘Langya virus’: is it a matter of concern?

**DOI:** 10.1080/01652176.2022.2117874

**Published:** 2022-09-01

**Authors:** Om Prakash Choudhary, Priyanka, Mathumalar Loganathan Fahrni, Asmaa A. Metwally, AbdulRahman A. Saied

**Affiliations:** a Department of Veterinary Anatomy and Histology, College of Veterinary Sciences and Animal Husbandry, Central Agricultural University (I), Aizawl, India; b Department of Veterinary Microbiology, College of Veterinary Science, Guru Angad Dev Veterinary and Animal Sciences University (GADVASU), Rampura Phul, India; c Faculty of Pharmacy, Universiti Teknologi MARA (UiTM) Puncak Alam Campus, Selangor, Malaysia; d Department of Surgery, Anesthesiology and Radiology, Faculty of Veterinary Medicine, Aswan University, Aswan, Egypt; e National Food Safety Authority, Aswan Branch, Aswan, Egypt; f Ministry of Tourism and Antiquities, Aswan Office, Aswan, Egypt

**Keywords:** Langya virus, monkeypox, Henipavirus, climate change, zoonotic, surveillance

Experts in infectious diseases have long cautioned that climate change and the destruction of nature will raise the likelihood of ‘zoonotic spillovers’, or the transmission of viruses from animals to people (Santana 2020). Recently, a new animal virus, called the Langya henipavirus (LayV), has been identified in humans in the Eastern China amid global efforts to combat the ongoing coronavirus disease 2019 (COVID-19) pandemic and monkeypox virus (MPXV) outbreak (Choudhary et al. 2022; Fahrni et al. 2022). Although the scientists have stated that the risk for spread among human beings is low, the virus is closely related to two other henipaviruses are known to infect people- Hendra virus and Nipah virus, which results in serious respiratory infections and can be lethal. Similarly, LayV can cause respiratory symptoms such as fever, cough, and fatigue, but none of the affected individuals have died to date.

This letter aims to highlight the possible impact of the LayV, assess the existent risk, and delineate the possible containment measures to mitigate the same. LayV virus belongs to the *Henipavirus* genus in the family *Paramyxoviridae*, which has a single-stranded RNA genome with a negative orientation. Prior to LayV, the genus Henipavirus included Hendra, Nipah, Cedar, Mojiang, and the Ghanaian bat virus. Fruit bats are the natural host for Hendra and Nipah virus (Drexler et al. 2012). The genomic analysis of the LayV genome has shown that the virus is closely linked to Mojiang henipavirus (Zhang et al. 2022), a rat-borne virus that was first identified in southern China, Yunnan province in 2012 after three miners developed severe pneumonia and died. The serum samples collected from the dead miners were sent to the Wuhan Institute of Virology that showed negative results for the Ebola virus, Nipah virus, and bat SARS-CoV Rp3. However, the fate of the miners indirectly revealed the isolation and identification of the Mojiang paramyxovirus (MojV) in rats (*Rattus flavipectus*) in China (Wu et al. 2014). It is reported that Mojiang henipavirus enters human cells using a mechanism different from those used by Hendra virus and Nipah virus (Rissanen et al. 2017). There is no evidence that the cedar virus, Ghanaian bat virus [now known as Kumasi virus (KV)], or Mojiang virus can infect humans yet. Hendra and Nipah, however, infect people. Evolutionary virologists believe that LayV is carried by shrews, which might have infected people directly or through an intermediate animal. They added that thus far and since 2018, only 35 people have been infected. None of the cases were apparently linked. LayV can present itself with fever, fatigue, cough, loss of appetite, and muscle aches. The common symptom found in those infected was a fever. The virus was the only potential pathogen found in 26 of the 35 people, suggesting that LayV was the cause of the febrile illness (The Guardian 2022).

While the LayV was first identified in the north-eastern provinces of Shandong and Henan in late 2018 (Figure 1), it was only formally identified by scientists on 4 August 2022 and described in length in the New England Journal of Medicine (Zhang et al. 2022). The virus was likely transmitted from animals (rodent-like mammals) to humans, and Taiwan’s health authority is now monitoring the spread at the China-Taiwan border. Evolutionary virologists tested wild animals and found LayV viral RNA in more than a quarter of 262 shrews tested. The finding suggested that the shrews may have been a ‘natural reservoir’ (Zhang et al. 2022). The virus was also detected in 2% of domestic goats and 5% of dogs. Although the transmission between people remains unclear, the majority of the 35 cases were in farmers and factory workers. Contact tracing of nine patients with 15 close-contact family members revealed no close-contact LayV transmission, but the study’s sample size was too small to determine the status of human-to-human transmission (The Guardian 2022). Because LayV belongs to a family of the closely related Henipaviruses, which includes the Nipah virus (NiV), it is of concern. The NiV infection varies from mild (asymptomatic infection) to severe (acute respiratory infection and fatal encephalitis, or brain swelling) disease or even death, with a case fatality rate of 40% to 75%. NiV infection can establish person-to-person spread in hospital settings (WHO 2018), but no person-to-person transmission has been reported with Hendra virus. However, should the LayV transmit from person-to-person so readily, it would be omnipresent.

**Figure 1.**
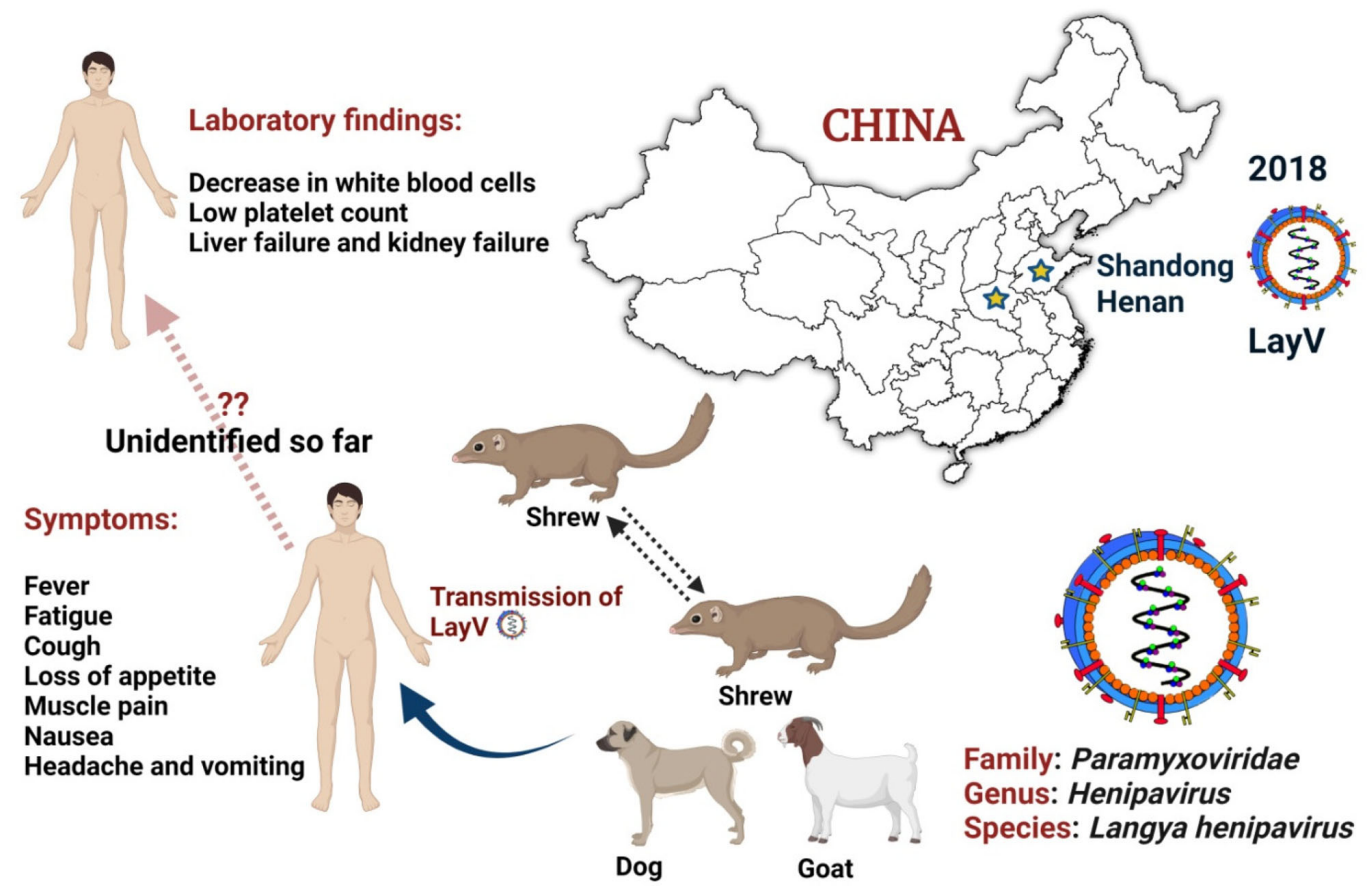
Emergence and zoonoses of Langya henipavirus. Langya henipavirus (LayV) has infected 35 people till so far in the eastern Chinese provinces of Shandong and Henan between April 2018 and August 2021. Strikingly, LayV has mostly infected farmers, with symptoms ranging from severe pneumonia to cough, and none of the cases seem to be linked. LayV viral RNA was identified in 27% of the 262 shrews studied and LayV antibodies were discovered in a few goats and dogs, suggesting that shrews are a reservoir for the virus.

Unsurprisingly, there aren’t any approved treatments or specific vaccines for henipaviruses, including the Langya virus, to date. Ribavirin may be a good option when there no other treatments are available for viral infections. Ribavirin is effective against the Hendra and Nipah viruses. These two might also be treated with chloroquine, a medication for malaria. Therefore, if necessary, these two treatments may also aid in controlling the Langya virus.

Scientists have estimated that 70% of newly emerging infectious diseases have been transmitted to humans through interaction with animals, a trend that has accelerated as the expanding human populations have a greater impact on wildlife habitats, especially viral pathogens (Saied et al. 2021). The climatic change also drives zoonotic epidemics by altering the host-pathogen interactions (Carlson et al. 2022; Rupasinghe et al. 2022). There is indeed an urgent need for the development of new vaccines for the re-emerged viral diseases and timely delivery of such vaccines to areas of high epidemiological risk (Khairi et al. 2022). Climate change is expected to exacerbate the alarming trends of zoonotic spillovers where animal viruses begin to infect humans, wherein more than half of the world’s human infectious illnesses are attributed to climate changes (Mora et al. 2022). It is imperative to conduct active surveillance in a transparent and globally collaborative manner in order to decrease the likelihood that an emerging virus would cause a health disaster. Further research is needed for questions which remain unanswered.

## Data Availability

Data sharing does not apply to this article as no datasets were generated or analyzed during the current study.
